# Supplementary material for: Patterns of Intron Gain and Loss in Fungi
Source: PLoS Biol. 2004 Nov 30;2(12):e422. doi: 10.1371/journal.pbio.0020422 (PMC532390; doi:10.1371/journal.pbio.0020422)
Supplement: Table S1 — Also available at http://genes.mit.edu/NielsenEtAl/. (4.3 MB ZIP). [file pbio.0020422.st001.zip › NielsenEtAl/html/906.html]

AN5343.1.NCU09730.1.MG01757.1.FG05088.1


```
 CLUSTAL W (1.82) Multiple Sequence Alignments - Introns Inserted


Sequence 1: MG01757.1	1070 aa
Sequence 2: FG05088.1	939 aa
Sequence 3: NCU09730.1	956 aa
Sequence 4: AN5343.1	966 aa
Alignment Length: 1118 aa
Number Identitical Residues: 571 aa
Alignment Score (without introns) 24279


MG01757.1 	--~---MSSANSIKV~VARFRPQNRVEIESGGQPIVKFDGDDTCTVD0SKEAQGSFTFDR
NCU09730.1	--~--MSSSANSIKV~VARFRPQNRVEIESGGQPIVTFQGPDTCTVD0SKEAQGSFTFDR
FG05088.1 	--~---MSSANSIKV~VARFRPQNKVELASGGTPIVSFNGDDTCSLN0SKEAQGSFTFDR
AN5343.1  	MA2SSTSNPSNTIKV1VARFRPQNKVELASGGQPIVEFENDETCSIN0SKEASGSFTFDR
          	 : ::  ..:*:*** ********:**: *** *** *:. :**::: ****.*******

MG01757.1 	VFDMSCKQSDIFDFSIKPTVDDILNGYNGTVFAYGQTGAGKSYTMMGTNIDDDDGRGVIP
NCU09730.1	VFDMSCKQSDIFDFSIKPTVDDILNGYNGTVFAYGQTGAGKSYTMMGTSIDDPDGRGVIP
FG05088.1 	VFDMGCKQQDIFDFSIRSTVDDILNGYNGTVFAYGQTGAGKSYTMMGTNIDDDEGRGVIP
AN5343.1  	VFPMDSKQTDVFNYSIAPTVDDILNGYNGTVFAYGQTGAGKSYTMMGSDIDDEVGKGIIP
          	** *..** *:*::** .*****************************:.***  *:*:**

MG01757.1 	RIVEQIFASILSSPSTIEYTVRVSYMEIYMERIRDLLAPHNDNLPVHEEKNRGVYVKGLL
NCU09730.1	RIVEQIFTSILSSAANIEYTVRVSYMEIYMERIRDLLAPQNDNLPVHEEKNRGVYVKGLL
FG05088.1 	RIVEQIFASIMSSPGTIEYTVRVSYMEIYMERIRDLLQPQNDNLPVHEEKNRGVYVKDLL
AN5343.1  	RIVEQIFASILTSPSNIEYTVRVSYMEIYMERIRDLLVPQNDNLPVHEEKSRGVYVKGLL
          	*******:**::*...********************* *:**********.******.**

MG01757.1 	EVYVSSVQEVYEVMRRGGNARAVAATNMNQESSRSHSIFVITITQKNVETGSAKSGQLFL
NCU09730.1	EIYVSSVQEVYEVMRRGGNARAVAATNMNQESSRSHSIFVITITQKNVETGSAKSGQLFL
FG05088.1 	EIYVSSVQEVYEVMRRGGNARAVAATNMNQESSRSHSIFVITITQKNVETGSAKSGQLFL
AN5343.1  	EVYVSSVQEVYEVMRRGGNARAVAATNMNQESSRSHSIFVITVTQKNLETGSAKSGQLFL
          	*:****************************************:****:************

MG01757.1 	VDLAGSEKVGKTGASGQTLEEAKKINKSLSALGMVINSLTDGKSSHVPYRDSKLTRILQE
NCU09730.1	VDLAGSEKVGKTGASGQTLEEAKKINKSLSALGMVINALTDGKSSHVPYRDSKLTRILQE
FG05088.1 	VDLAGSEKVGKTGASGQTLEEAKKINKSLSALGMVINALTDGKSSHIPYRDSKLTRILQE
AN5343.1  	VDLAGSEKVGKTGASGQTLEEAKKINKSLSALGMVINALTDGKSTHIPYRDSKLTRILQE
          	*************************************:******:*:*************

MG01757.1 	SLGGNSRTTLIINCSPSSYNDAETLSTLRFGMRAKSIKNKAKVNAELSPTELKQLLGKAR
NCU09730.1	SLGGNSRTTLIINCSPSSYNDAETLSTLRFGMRAKSIKNKAKVNAELSPAELKQMLAKAK
FG05088.1 	SLGGNSRTTLIINCSPSSYNDAETLGTLRFGMRAKSIKNKAKVNAELSPAELKSLLKKAQ
AN5343.1  	SLGGNSRTTLIINCSPSSYNDAETVSTLRFGVRAKAIKNKAKVNAELSPAELKQLLRRAQ
          	************************:.*****:***:*************:***.:* :*:

MG01757.1 	TQITSFEKYIVSLEGEVQLWRSGETVPKDKWVPARSADGTAPATKGALPARPATPSRLVA
NCU09730.1	TQITSFENYIVNLESEVQVWRGGETVPKEKWVPP-LELAITPSKSASTTARPSTPSRLLP
FG05088.1 	GQVTNFESYISNLEGEIQLWRAGEAVPKEKWATPAASTDVVTRTKADAARTSTRPSTPSL
AN5343.1  	SQVTSFENYISALESEVSSWRSGETVPREKWTPARNAEAVGAAKAEARGPRPSTPSRLHD
          	 *:*.**.**  **.*:. **.**:**::**...       . .       .: **    

MG01757.1 	ETASRSETP--ALTERSGTPSIPLEKDEREEFLRRENELQDQLAEKESQATNAERQVREM
NCU09730.1	E--SRAETP--AISDRAGTPSLPLDKDEREEFLRRENELQDQIAEKESIAAAAERQLRET
FG05088.1 	ISDSRSETP--AISERAGTPSLPLDKDEREEFLRRENELQDQISEKESQTVAAEKQLRET
AN5343.1  	VSRSETPRPDSRIGDRSSTPSIVLEKDEREEFLRRENELQDQIAEKESHIATIERGLREA
          	 : *.:  *.: : :*:.***: *:*****************::****  .  *: :** 

MG01757.1 	KEELTIIKEHDGKLGKENERLMSDHNELKLQFERINFESKEAQITMDALKEANAELTTEL
NCU09730.1	KEELIALKDHDSKLGKENERLISESNEFKMQLERLAFENKEAQITIDGLKDANSELTAEL
FG05088.1 	KEELAYLKDHDSKVGKENEKLTTEVNEFKMQLERLTFESKEAQITMDALKEANSELTTEL
AN5343.1  	RDELRTLKENSARSGKDNEKLNAEVNELRMHLEKVSYESKEAAITMDSLREANSELTAEL
          	::**  :*::..: **:**:* :: **:::::*:: :*.*** **:*.*::**:***:**

MG01757.1 	DELKQQLLDVKMNAKESGAALDEKEKRKAEKMAKMMAGFELGADVFSDNEKSIADAIKQL
NCU09730.1	DEVKQQMLDMKMSAKETSAVLDEKEKKKAEKMAKMMAGFDLSGDVFSDNERAVADAIAQL
FG05088.1 	DDMKQQLLDVKMSAKENGAALDEKERRKAEKMAKMMAGFDLGGDVFSENERHIAETIEKV
AN5343.1  	DEVKQQLLDVRMKAKETTAALDEKEKKKAEKMAKMMAGFDLGGEVFSDNERKLQNLIQRV
          	*::***:**::*.***. *.*****::************:*..:***:**: : : * ::

MG01757.1 	EALQELSNSGDPIPPDELKALQSKLVATQGIVRQAELSLYS-SASSGSEARRRVELEQRL
NCU09730.1	DALFEISSAGDAIPPEDIKALREKLVETQGFVRQAELSSFS-AASSDAEARKRAELEARL
FG05088.1 	DSLHELSATGDNIAPDEFKALRSRLVETQGIVRQAELSMYS-TTSGDADSRRRQELEARL
AN5343.1  	DSLHQIAEAGEVIAPDDLLELRASLSETQGFIRQAELTVNDRSELNVLQDSRRMELEQKL
          	::* ::: :*: *.*:::  *:  *  ***::*****:  . :  .  :  :* *** :*

MG01757.1 	DTLQQEYEDLLSRNLGEGDVEELRSRLEKAFTDKQGAQTDLVDDLKAEIERKTAEAERMK
NCU09730.1	EALQQEHEELLSRNLTEADKEEVKALLAKSLSDKSAVQVELVEQLKADIALKNSETEHLK
FG05088.1 	EAVQAEYEEILTRNLGPEDVEEVKARLENAFANRQTAQSAFVDELKADIAQKAAENTRMK
AN5343.1  	ANLEQDYESLLARNLGEEDLAEIKERLEKVYIAKKDVELAAASELQQQLARKDEELTKLQ
          	  :: ::*.:*:***   *  *::  * :    :. .:   ..:*: ::  *  *  :::

MG01757.1 	VLIEDLQRQAKASGANGNAGVSPVMNGKSVAQQMAEFDVMKKSLMRDLQNRCERVVELEI
NCU09730.1	ALVDDLQRRVKAGG-----AGVAMANGKTVQQQLAEFDVMKKSLMRDLQNRCERVVELEI
FG05088.1 	TLIDDLQQRVKAG------ATAPMANGKTIQQQIAEFDVMKKSLMRDLQNRCERVVELEI
AN5343.1  	QSLADSQSRGSTNG----------AASKSLQQQIAEFDAMKKSLMRDLQNRCERVVELEI
          	  : * * : .:..            .*:: **:****.*********************

MG01757.1 	SLDETREQYNNVLRSSNNRAQQKKMAFLERNLEQLTQVQRQLVEQNSSLKKEVAIAERKL
NCU09730.1	SLDETREQYNNVLRSSNNRAQQKKMAFLERNLEQLTQVQRQLVEQNSALKKEVAIAERKL
FG05088.1 	SLDETREQYNNVLRSSNNRAQQKKMAFLERNLEQLTQVQRQLVEQNSALKKEVAIAERKL
AN5343.1  	SLDDAREQYNNVLRSSNNRAQQKKMAFLERNLEQLTHVQRQLVEQNSSLKKEVAIAERKL
          	***::*******************************:**********:************

MG01757.1 	IARNERIQSLESLLQDSQEKMAQANHK2----~---------------------------
NCU09730.1	MARNERIQSLESLLQESQEKMAQANHK~YVFT~PLLLLHALIIIS-----------ISAN
FG05088.1 	IARNERIQSLESLLQDSQEKMAAANHK~YARP2---------------------------
AN5343.1  	IARNERISSLEALLQESQEKLTQANHR~YACT~FYSQEIPSLEIQEQSLFYKVPSQKKHI
          	:******.***:***:****:: ***:    .       .    ...:   . .:. .  

MG01757.1 	--------FEMQLASVKERLEAAKAGSTRGIGSPTGLGGFSFTNAGSRIAKPLRGGGGGT
NCU09730.1	LPSSYCTRFEVQLAAVKDRLEAAKAGSTRGLGTDAGLGGFSIG---SRIAKPLRGGGDAV
FG05088.1 	--------FEVQLAAVKERLELAKAGSTRGLNSP---GGFSFANAGSRIAKPLRGGGGGN
AN5343.1  	LTSTPNNSFEAQLTAVKERLEAAKQGSTKGLPGMDSNGGFSFAG--SRIAKPLRGGGGGN
          	 .::  . ** **::**:*** ** ***:*:    . ****: .  ***********.. 

MG01757.1 	D-NTAANPAISNLQSSDNKRSS0IQPCSNRAPRRKRAISSGLTNKNPTSVLAAVRLENNH
NCU09730.1	AGATATNPTIATLQQNP-----~------------------PENKRSSWFFQKS------
FG05088.1 	D-AAPSIPTIQNLQGQN-----~-----------EGNISSGSSSKRASWFFAKS------
AN5343.1  	E-------PVAAVQSQD-----~------------------TGTKRTSWFFDRR------
          	        .:  :* .                           .*..: .:         

MG01757.1 	LDRNYEADNDHQGDNDKESLEKHLDDPDPRLTVQAPDGIADRKVLSAAQLRRSDTTAKRT
NCU09730.1	------------------------------------------------------------
FG05088.1 	------------------------------------------------------------
AN5343.1  	------------------------------------------------------------
          	                                                            

MG01757.1 	IKGDDCPFLLREQAGLHKGEGDKGRQADDKHEGGKNQEGEDSLA
NCU09730.1	--------------------------------------------
FG05088.1 	--------------------------------------------
AN5343.1  	--------------------------------------------
          	
```
